# Novel fusion antigen displayed-bacterial ghosts vaccine candidate against infection of Escherichia coli O157:H7

**DOI:** 10.1038/srep17479

**Published:** 2015-12-02

**Authors:** Kun Cai, Wei Tu, Yuenan Liu, Tao Li, Hui Wang

**Affiliations:** 1 Department of Microbiology, Anhui Medical University, Hefei, 230032, PR China; 2 State Key Laboratory of Pathogens and Biosecurity, Beijing Institute of Microbiology and Epidemiology, No. 20 Dongdajie, Fengtai District, Beijing 100071, PR China.

## Abstract

Infection with *Escherichia coli* O157:H7 may develop into hemorrhagic colitis, or hemolytic uremic syndrome (HUS), which usually causes kidney failure or even death. The adhesion and toxins are the important virulent factors. In this study, a novel vaccine candidate rSOBGs was constructed based on the bacterial ghost (BG). rSOBGs maintained the integrity of cellular morphology and displayed the linear Stx2Am-Stx1B antigen on the surface of outer membrane. rSOBGs induced Stxs-specific IgA/IgG antibodies and stronger intimin-specific IgA/IgG antibodies effectively in sera in this study. *In vivo*, the rSOBGs provided the higher protection rate (52%) than native bacterial ghost-OBGs (12%) when challenged intragastricly with high dose (500 LD50) viable *E. coli* O157:H7. Meanwhile, the rSOBGs provided higher protection rate (73.33%) than OBGs when challenged with 2 LD50 even to 5 LD50 lysed *E. coli* O157:H7. *In vitro*, the rSOBGs-immunized sera possessed neutralizing activity to lysed pathogenic bacteria. Furthermore, the results of histopathology also displayed that the administration of rSOBGs have the ability to reduce or inhibit the adhesion lesions and toxins damages of organs. The novel vaccine candidate rSOBGs induced both anti-toxin and anti-adhesion immune protection, suggesting the possibility to prevent the infectious diseases caused by *Escherichia coli* O157:H7.

*Enterohemorrhagic Escherichia coli* (EHEC) O157:H7 is a food-borne pathogen, could cause diarrhea, hemorrhagic colitis, thrombotic thrombocytopenic purpura and HUS in humans^1^,^2^, especially in young children and the elderly (fatality rate: 5 to 10%)^3^,^4^. *Escherichia coli* (*E. coli*) O157:H7 characteristically results in attaching and effacing (A/E) lesions. Intestinal colonization of pathogenic bacteria and release of Shiga toxins are two important factors in infection of Enterohemorrhagic Escherichia coli.

*E. coli* O157:H7 produces one or both of two types of Shiga toxins (Stxs) which are responsible for HUS and are categorized into two distinct groups, Stx1 and Stx2. The Stxs are complex holotoxins within the basic 1A:5B structure^5^,^6^. The A and B subunits of Stx1 and Stx2 are 68% and 73% similar at the amino acid level^7^. Despite the degree of homology, Stx1 and Stx2 are reported to be immunologically distinct^8^. Wen *et al*. and others have demonstrated genetic toxoids of Stxs protect mice against homologous but not heterologous toxin challenge^9^,^10^. A hybrid StxA2/StxB1 holotoxoid^11^ and a genetic Stx2B-Stx1B^12^ could elicit neutralizing antibody response, and a safer and higher production of genetic toxoid SAmB which could induce cross-neutralizing antibodies has been found recently^13^.

Some surface proteins, including intimin, are important antigens which give bacteria the ability to adhere tightly to host cells^14^,^15^,^16^,^17^. It can be assumed that vaccine-induced antibodies against these surface antigens would effectively hamper the adherence of the challenge bacteria to the target cells^18^,^19^. Bacterial ghost (BG) is produced by the expression of PhiX174 lysis gene E, and results in cellular lysis and cytoplasmic loss^20^. BG maintains the cellular morphology and native surface antigenic structure^21^, and posses the adjuvant property^22^. The effects of BGs vaccines have been demonstrated in various pathogens, such as *Vibrio cholerae*^23^, *Pasteurella haemolytica*^24^ and *Helicobacter pylori*^25^, and the BG has been developed to be the candidate vaccine against the infection of *E. coli* O157:H7^26^,^27^.

It is considered that co-infection of viable *E. coli* O157:H7 organism and toxins is general, and a novel vaccine which could provide the co-prevention of bacterial adhesion and toxic damage is imminent. In this study, the linear Stx2Am-Stx1B antigen was displayed on the surface of *E. coli* O157:H7 BGs based on a sandwich vector pSOmpA^28^,^29^,^30^,^31^ which was constructed by the partial out membane protein A (OmpA) of *Shigella dysenteriae* and partial OmpA of *E. coli* to construct a novel candidate vaccine named rSOBGs. The immunogenicity, protection ability and immunologic mechanism against the challenge of *E. coli* O157:H7 were described subsequently.

## Materials and Methods

### Bacterial strains, plasmids, cell lines and media

Bacteria were grown in Luria-Bertani (LB) broth or agar (Oxoid) supplemented with 100 μg/ml of ampicillin for selection of recombinant plasmid. LB broth or agar without ampicillin was used for culturing *E. coli* O157:H7. Hep-2 cells and Vero cells were conserved in the laboratory, and the cells were grown in Dulbecco’s modified eagle medium (DMEM) supplemented with 10% (v/v) fetal bovine serum (FBS).

### Ethics statement

The Ethics of Animal Experiments of Beijing Institute of Microbiology and Epidemiology approved the study. The all experiments and methods were carried out in accordance with the approved relevant guidelines. BALB/c mice were maintained in the Animal Center under specific pathogen-free conditions in Beijing Institute of Microbiology and Epidemiology. BALB/c mice were fed with standard diet and water, maintained under the following conditions: 12 h light/12 h dark controlled lighting, 24 °C to 28 °C temperature, and 55% relative humidity. All animals were handled under the care and supervision of a veterinarian. The mice that were severely injured by E. coli O157:H7, were sacrificed by cervical dislocation at the end of experiment.

### Construction of expression plasmid pOSAmB

The 1–233 amino acids of OmpA (Genbank: NC_007606) amplified from *Shigella dysenteriaewas* 51054 strain (primers: SOup/SOdown, table S1), and the 234–325 amino acids of OmpA amplified from *E. coli* O157:H7 EDL 933 strain (primers: EOup/EOdown). Splicing overlap extension (SOE) PCR was introduced to generate SOmpA gene with primers SOdown/EOup (overlaid) and flanking primers SOup/EOdown. Subsequently, primers Enzup/Enzdown (overlaid) and flanking primers SOup/EOdown were used to introduce two restriction enzyme sites into SOmpA to generate ESOmpA gene. The ESOmpA gene was ligated into pMD18-T Simple vector to construct pESOmpA for sequencing, then plasmid was digested by EcoR I/Sac I, and the vector was harvested. SAmB gene^13^ was amplified from pSAmB (primers Stxup/Stxdown), and restriction enzyme sites EcoR I/Sac I were introduced. Thereafter, the gene was cloned into pMD18-T Simple vector to generate pESAmB for sequencing, and the generated SAmB gene was ligated into digested pESOmpA vector, and the plasmid was digested by XmaI and Nco I to generate a gene named OSAmB. This OSAmB gene was ligated into the expression vector pGEX to generate the expression plasmid pOSAmB.

### Preparation of rSOBGs

**Figure 1:**
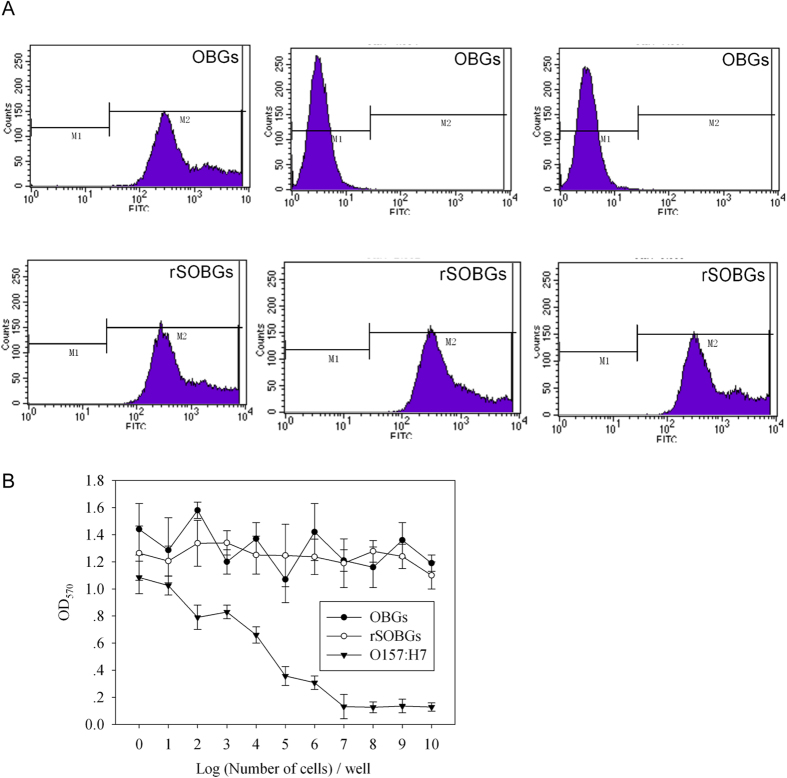
Verification of the surface antigens and evaluation of the cytotoxicity. (**A**). Verification of the surface antigens. Flow cytometry was adopted to detect 3 × 10^4^ Hep-2 cells. BGs were used as antigens, PBS contains chicken anti-Stx1B IgY, rabbit anti-Stx2A sera or rabbit anti-intimin sera was used as first anitibody, respectively. HRP labeled anti-chicken IgG or anti-rabbit IgG was used as test antibody according to their sources. (**B**). Cytotoxicity analysis of rSOBGs. Serial diluted BGs were incubated with Vero cell monolayer for 36 h, and the MTT assay was performed to detect the cytotoxicity. *P < 0.01 vs PBS group.

The correct pOSAmB plasmids were transformed into O157:H7 EDL933 as novel competent cells. The lysis plasmid pLysE (supp.Fig.1) was transformed into these novel competent cells to construct the vaccine strain of rSOBGs. Grown up to OD_600_ 0.3, the cultures of rSOBGs were induced by isopropyl β-D-thiogalactopyranoside (IPTG) at a final concentration of 1 mM at 28 °C. The induction of lysis was achieved by shifting the temperature from 28 °C to 42 °C when the OD_600_ reached 0.6, and the procedure was monitored by the optical densities. The lysis rate based on clony-forming unit (CFU) and morphous of rSOBGs were detected as described previously^27^. Subsequently, the rSOBGs were treated with distilled water with streptomycin to kill the survival bacteria. O157:H7 EDL933 strain without pOSAmB plasmid were prepared to BGs as control (named OBGs).

### Analysis of antigens by FACS

PBS contains chicken anti-Stx1B immunoglobin Y (IgY), rabbit anti-Stx2A sera^32^ or rabbit anti-intimin sera^33^ was incubated with rSOBGs for 1 h, respectively. Then, the FITC-labeled rabbit anti-chicken IgG or goat anti-rabbit IgG was added and incubated for another 1 h as second antibody according to the first antibody, respectively. After processing, the labeled rSOBGs were incubated with 10^6^ Hep-2 cells for 4 h. Finally, these cells were harvested and re-suspended with FACS buffer, and 3 × 10^4^ Hep-2 cells were detected by flow cytometry. OBGs cells were used as control.

### Analysis of cytotoxicity by MTT

Cytotoxicity assay of rSOBGs was performed by MTT assay on Vero cells. Serial diluted (1:10) rSOBGs were applied on Vero cell monolayer and incubated at 37 °C (CO_2_) for 36 h. Then, 100 μl/well 0.5 mg/ml 3-(4,5-Dimethylthiazol-2-yl)- 2,5-diphenyl tetrazolium bromide were added and incubated for another 4 h to produced formazan. The produced formazan were re-suspended by Dimethyl sulphoxide (DMSO, 150 μl/well), and the absorbance at 570 nm was detected. The OBGs and *E. coli* O157:H7 were used as controls.

### Mice immunization and challenge

Three-week-old male BALB/c mice (Vivarium of Academy of Military Medical Sciences, Beijing, China) were divided into groups randomly (Table S2). The mice were intragastricly administrated with 0.1 mg (corresponding to 10^8^ CFU) rSOBGs, OBGs or PBS which were re-suspended in 100 μl PBS on day 0 for primary and on day 14 for boost vaccinations, respectively. Different schemes for challenge were performed 14 days after the last immunization. 1. 25 mice from each group were intragastric challenge of 100 times 50% lethal doses (LD50, 10^9^ CFU) viable *E. coli* O157:H7 EDL933; 2. 25 mice from each group were intragastric challenge of 500 LD50 viable *E. coli* O157:H7 EDL933; 3. 15 mice from each group were intraperitoneal injection with 2 LD50 (5 × 10^8^ CFU) lysed *E. coli* O157:H7 88321; 4. 15 mice from each group were intraperitoneal injection with 5 LD50 lysed *E. coli* O157:H7 88321. The mice in scheme 1 and 2 (challenged with viable pathogenic bacteria) were deprived of food and water for 24 h and injected i.p. with Mitomycin (2.5 mg/kg).

In addition, 15 mice from each group were used to collect serum and irrigating solution of intestinal tract on days 0, 7, 14, 21 and 28 (3 mice were adopted for each time point) post the last immunization to determine the levels of specific antibodies. Surplus 10 mice from each group were used to collect the sera 14 days post the last immunization to perform the neutralization test *in vitro*. All remained mice were killed on day 21 post challenge.

### Detection of specific antibodies

The antibodies of serum and irrigating solution specific to OBGs, intimin, Stx1 and Stx2 were measured by enzyme linked immunosorbent assay. 100 μl OBGs (0.1 mg/ml), purified intimin protein, Stx1 or Stx2 (0.1 mg/ml) were coated in 96-well plates overnight at 4 °C, respectively. Goat anti-mouse IgA-HRP (Sigma, 1:5000) or goat anti-mouse IgG-HRP (Sigma, 1:5000) used as the detection antibodies. The reactions were developed with TMB and stopped with 2 M H_2_SO_4_. The absorbance at 450 nm was detected.

### Examination of histopathology

The dead and survival mice in rOBGs group which were used for challenge were sacrificed and subjected to a full necropsy. Tissue specimens (liver, kidney and intestine) were collected for histologic examination. The specimens were fixed in 10% buffered neutral formalin and processed via standard procedures. Sections (5 μm thickness) of paraffin-embedded tissues were stained with hematoxylin and eosin (H&E), and examined under optical microscopy.

### Neutralization test

The sera (100 μl) of mice immunized with different antigens (rSOBGs, OBGs and PBS, 10/group) were incubated with 2 LD50 and 5 LD50 lysed *E. coli* O157:H7 88321 (100 μl) at 37 °C for 1 h, respectively. These samples were injected into (i.p.) 7-weeks-old male BALB/c mice (10/group), and the mice were observed 14 days. All mice were coded to assess without bias.

### Statistical analysis

The results are presented as means ± standard error (S.E). Statistical significance was determined by ANOVA or Kruskal-Wallis test for parametric or nonparametric data, respectively. Mice survival differences were done by one sided Fisher’s exact test. *P* values < 0.05 were considered significantly.

## Results

### rSOBGs expressed the linear Stx2Am-Stx1B fusion antigen on the surface of outer membrane

The expression plasmid pOSAmB containing 2300 bp OSAmB gene was constructed, and the co-transformation of plasmids pOSAmB and pLysE generated the vaccine strain of rSOBGs successfully.

The OD_600_ reduced constantly after the shift of temperature. The precipitation 1 h after induction was harvested to evaluate the lysis rate, and it was counted as 99.99% ± 0.01%.

Outer membrane protein intimin could be confirmed in both rSOBGs and OBGs by flow cytometry, but the Stx2A and Stx1B were detected only on the surface of rSOBGs (Fig. 1A).

### rSOBGs were safe on cells model

At 10^7^ CFU, the pathogenic bacteria killed nearly 100% Vero cells. In contrast, no obvious cytotoxic effect were detected when the Vero cells were treated with 10 mg (equivalent to 10^10^ CFU of bacteria) rSOBGs or OBGs (*P* > 0.05) (Fig. 1B).

### Immunization with rSOBGs induced various specific antibodies efficiently

Samples collected on days 0, 7, 14, 21 and 28 were detected. As same as our previous study (27), the specific antibodies induced by OBGs and rSOBGs increased persistently. In this manuscript, only the data on day 28 were displayed.

**Figure 2:**
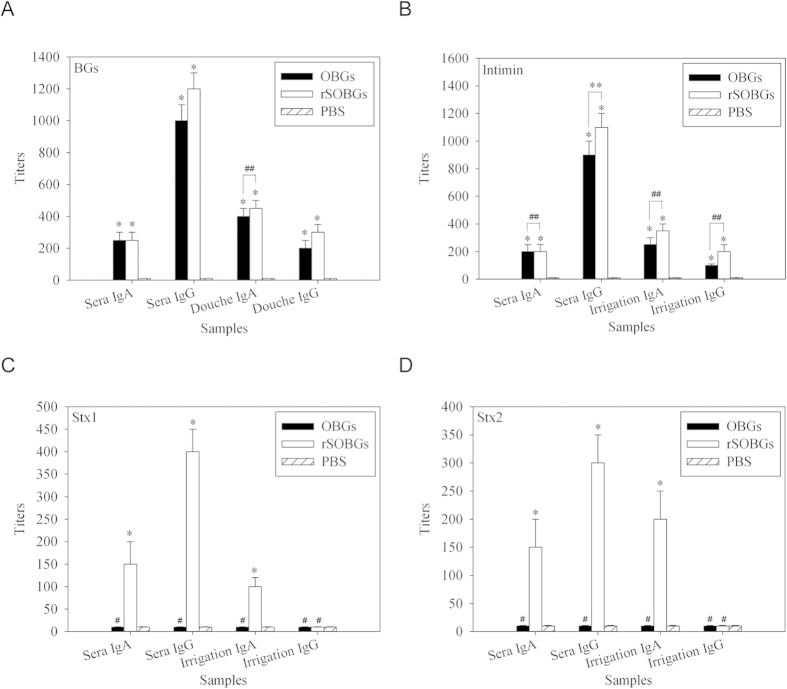
Detection of the specific IgA and IgG titers in sera and irrigating solution of immunized mice. ELISA was used to detect the levels of specific antibodies. OBGs, intimin, Stx1 or Stx2 was coated as antigen, respectively. Serial diluted serum or irrigating solution in PBS were added and incubated. HRP labeled anti-mouse IgA or IgG was used as the test antibody, respectively. (**A**). BGs-specific IgA and IgG antibodies in sera and irrigating solution. (**B**). intimin-specific IgA and IgG antibodies in sera and irrigating solution. (**C**). Stx1-specific IgA and IgG antibodies in sera and irrigating solution. (**D**). Stx2-specific IgA and IgG antibodies in sera and irrigating solution. *P < 0.01 vs PBS group; **P < 0.01; ^#^*P* > 0.05 vs PBS group; ^##^*P* > 0.05.

The antibodies specific to BGs were tested at first. IgA and IgG antibodies specific to BGs were elicited both by rSOBGs and OBGs (*P* < 0.01), and the levels of titer were similar (*P > *0.05) (Fig. 2A). No specific antibodies were detected in PBS group.

The antibodies specific to intimin, one of the most important factor in adhesion of EHEC, were tested subsequently. In contrast to PBS, both of the rSOBGs and OBGs could induce significant intimin-specific IgA/IgG antibodies (*P* < 0.01), and only one difference of IgG titers between rSOBGs and OBGs was detected in irrigation solution (*P* < 0.01) (Fig. 2B).

The effect to induce Stx1-specific or Stx2-specific IgA/IgG antibodies will be the unique advantage of rSOBGs against OBGs in our expectation. In contrast to the OBGs and PBS, immunization of rSOBGs stimulated significant specific IgA antibodies against Stx1 and Stx2 in sera and irrigating solution (*P* < 0.01), and the specific IgG antibodies against Stx1 and Stx2 were detected only in sera (*P* < 0.01) (Fig. 2C,D).

### rSOBGs provided stronger and cross protection

rSOBGs not only displayed stronger protection against the viable *E. coli* O157:H7, but also displayed cross protection against the lysed *E. coli* O157:H7.

**Figure 3:**
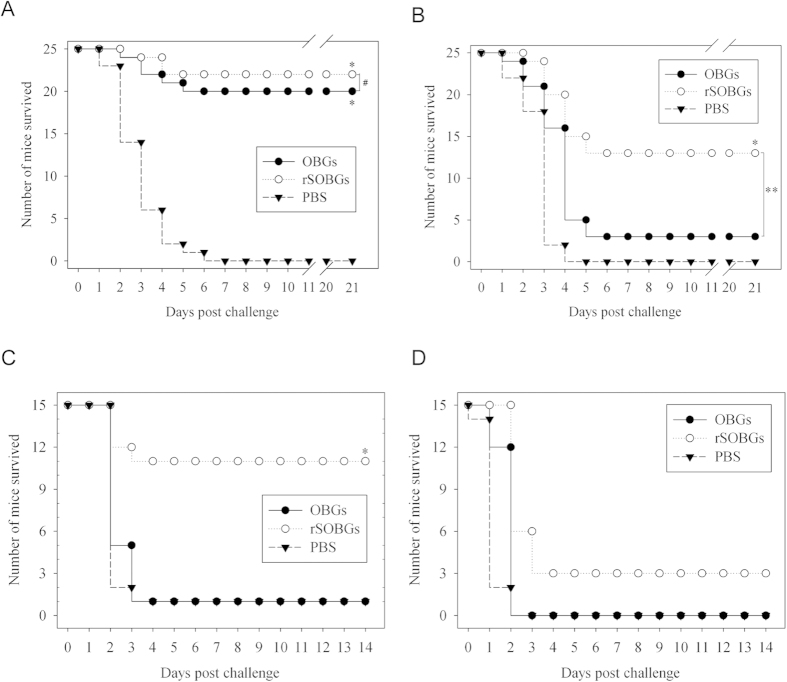
Protection of rSOBGs-immunized mice against lethal dose challenge of viable and lysed O157:H7. The male BALB/c mice immunized on day 0 for primary immunization, and immunized on day 14 for boost. 14 days post the last immunization, the mice were challenged. (**A**). Mice were intragastric challenged with 10^9^ CFU viable O157:H7 EDL933. (**B**). Mice were intragastric challenged with 5 × 10^9^ CFU viable O157:H7 EDL933. (**C**). Mice were challenged i.p. with 2 × 10^8^ CFU lysed O157:H7 88321. (**D**). Mice were challenged i.p. with 5 × 10^8^ CFU lysed O157:H7 88321. *P < 0.01 vs PBS group; **P < 0.01; ^##^*P* > 0.05.

rSOBGs provided the similar (*P* > 0.05) protection rate (22/25, 88%) with the OBGs (20/25, 80%) when the challenge dose was 100 LD50 (Fig. 3A). When the intragastric challenge dose was lifted to 500 LD50, the protection rate of OBGs (3/25, 12%) decreased sharply, and the rSOBGs provided higher protection rate (13/25, 52%) than that of OBGs (*P* < 0.01) (Fig. 3B). No mice survived in PBS groups when challenged either the high or the low dose.

No mice survived in PBS groups after the challenge. The rSOBGs provided higher protection rate (11/15, 73.33%) than that of OBGs (*P* < 0.01) when challenge with 2 LD50 lysed *E. coli* O157:H7 88321 (Fig. 3C). When the dose upgraded to 5 LD50, 3 mice could survive in rSOBGs group but no survival in OBGs group (Fig. 3D).

### Administration of rSOBGs reduced the damage of organs

**Figure 4:**
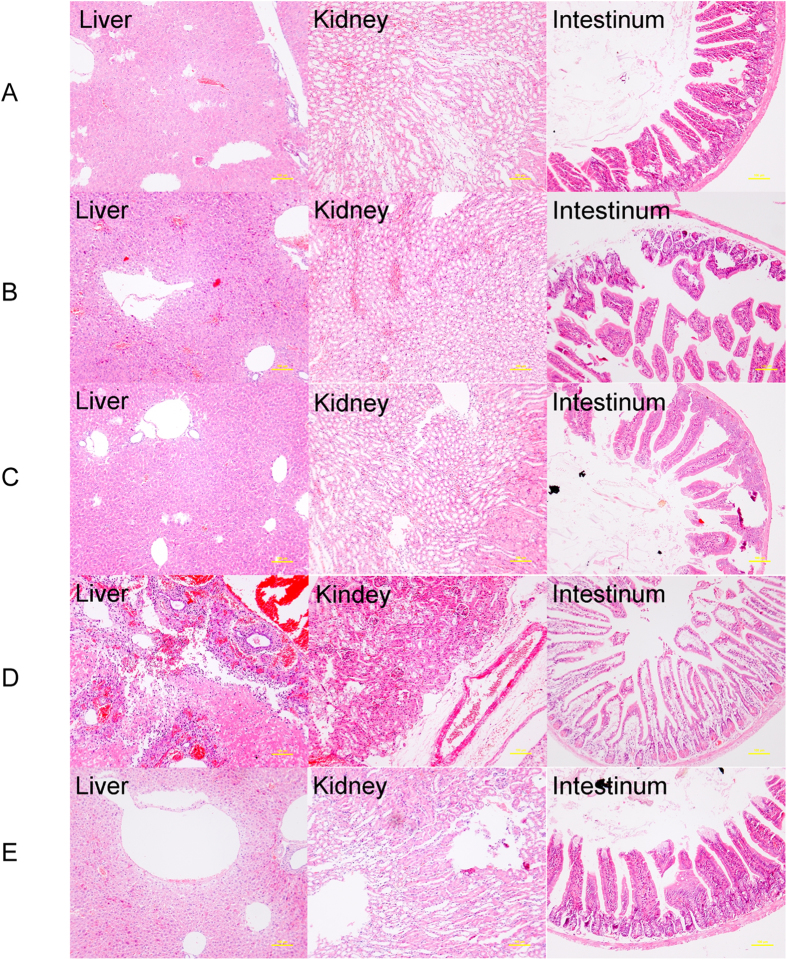
Pathology of liver, kidney and intestine of rOBGs immunized mice. Sections of paraffin-embedded tissue were stained with hematoxylin and eosin, and examined under light microscopy. Pathological changes were labeled by arrows. (**A**). Normal mice. (**B**). Mice succumbing to intragastric challenge with viable O157:H7 EDL933. (**C**). Survival mice intragastric challenged with viable O157:H7 EDL933. (**D**). Mice succumbing to challenge with lysed O157:H7 88321. E. Survival mice challenged i.p. with lysed O157:H7 88321.

The intragastric challenge with viable *E. coli* O157:H7 EDL933 caused the major injury in intestine (epithelial cells necrosis), mainly accompanied with the damage of kidney, and no obvious damage was detected in liver (Fig. 4B). The immunization of rSOBGs reduced the damage in intestine efficiently, and the symptom in kidney vanished (Fig. 4C). The intraperitoneal injection with lysed *E. coli* O157:H7 88321 damaged the kidney (glomcrulus hyperaemia and death) and liver (focal necrosis and mesenchyme hyperaemia), but not the intestine (Fig. 4D). The administration of rSOBGs relieved these symptoms enormously (Fig. 4E). The normal mice were used as control (Fig. 4A).

The pathological changes of dead mice between OBGs group and rOBGs group were identical, and all of the survival mice showed negative serious pathological damage (data not shown).

### rSOBGs immune sera possessed neutralizing ability against lysed *E. coli* O157:H7

**Figure 5:**
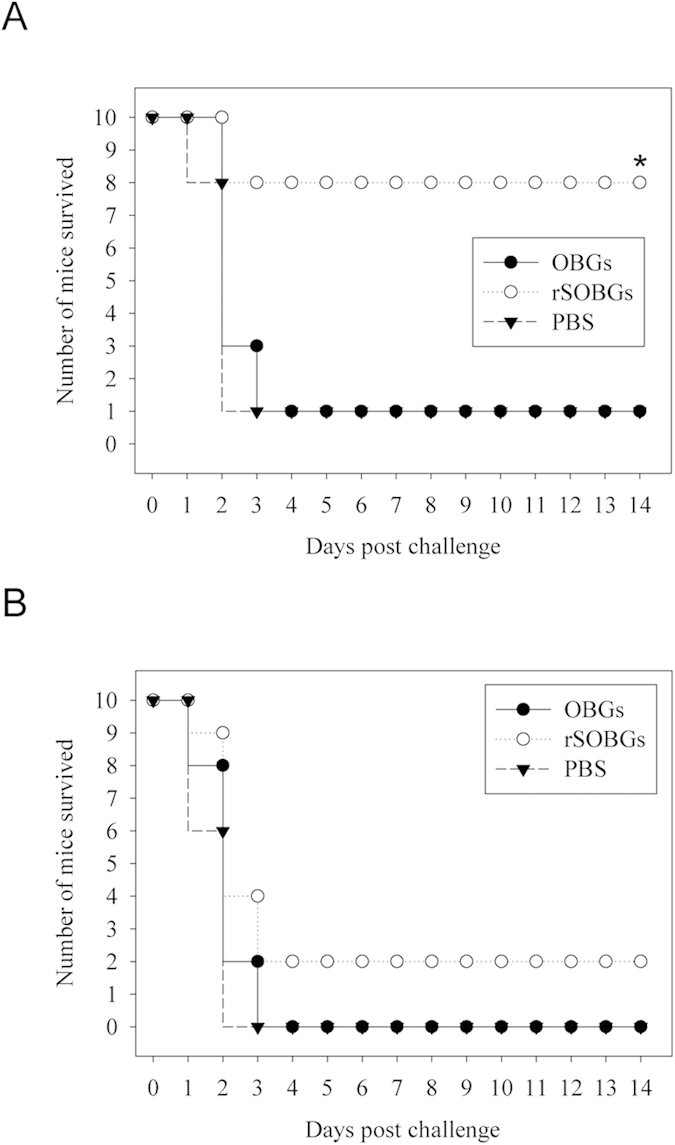
*In vitro* protection of antisera against lysed O157:H7. The antisera were incubated with different lethal dose lysed O157:H7 88321, then these samples were challenged i.p. in BALB/c mice. The survival was observed for 14 days. (**A**). The neutralizing capacity against 2 × 10^8^ lysed O157:H7 88321 *in vitro*. (**B**). The neutralizing capacity against 5 × 10^8^ lysed O157:H7 88321 *in vitro*. **P < 0.01 vs PBS group.

To test the neutralizing ablity of rSOBGs immune sera, it was mixed with lysed pathogen and injected i.p. mice . Ten percent mice (1/10) survived in the OBGs and PBS groups with 2 LD50 lysed *E. coli* O157:H7 88321. The rSOBGs provided significantly higher protection (80%, 8/10) than that of OBGs (10%, 1/10) (Fig. 5A). When the dose of pathogenic bacteria added to 5 LD50, all mice died in OBGs and PBS groups, but 2 mice(20%, 2/10) in rSOBGs group still survived (Fig. 5B).

## Discussion

The 325 residue OmpA protein has been shown by physical analysis that N-terminal half of the molecule forms a β-barrel structure within the hydrophobic membrane layer that supports four loops (L1–L4) of amino acids at the outer surface, which may serve as OmpA receptor sites for the attachment of various phages and colicins^34^,^35^,^36^. In a previous study, a sandwich vector pHS64 which contained a recombinant (*S. dysenteria* and *E. coli*) OmpA had been constructed^37^. The OmpA sequence of *S. dysenteria* encodes five additional codons in L3 than *E. coli*, and these additinal codons are beneficial to the folding of foreign proteins. Some proteins, such as hemagglutinin^38^, VP1^39^, malarial’s SERP antigen^40^, and the HBcAg-149^41^ had been displayed in L3 by this vector.

rOSBGs could induce Stxs-specific antibodies in sera due to the surface expression of Stxs antigens, but failed to induce Stxs-specific IgG antibodies in irrigating solution, the interruption of intestinal mucosa against IgG antibody^42^ and the low titer of IgG antibody may be the reseaons. The Stxs-specific antibodies could neutralize the Stxs, and the antisera of rOSBGs could provide effective cross protection against the challenge of low lethal dose lysed bacteria which contained two subtypes of Stxs. While this protection was locked into a narrow range of lethal dose challenges compared to the toxoid SAmB vaccine^13^. The decreased protection rate when challenged with high lethal dose may due to the low titer of the Stxs-specific antibodies which were induced by the insufficient expression of toxoid on the surface of *E. coli* O157:H7. The co-delivery of toxoid which were encapsulated by the BGs will be a beneficial attempt.

The intimin is the integrated membrane component on the surface of the *E. coli* O157:H7, and the levels of intimin specific antibodies hase been demonstrated to be the same between OBG and rSOBG due to the identical membrane structure. In addition, a higher intimin-specific IgG antibody was induced by rOSBGs than OBGs in sera irrigation solution. The adjuvant property of Stx1B may be a potential reason^43^. The promotion of adhesion between O157:H7 and target cells by Stx2^44^,^45^ may be anorther reason. We presumed that the expression of SAmB may promote the adhesion between rSOBGs and enterocytes, more intimin could be displayed as antigens, and the higher titers of antibodies could be induced.

For the protection rate against viable *E. coli* O157:H7, although no significant difference was found between rOSBGs group and OBGs group when challenged with low dose (100 LD50) viable *E. coli* O157:H7, but the rOSBGs provided stronger protection than OBGs when challenged with 500 LD50 viable *E. coli* O157:H7. The adjuvant property of Stx1B, the adhesion promoting effect of Stx2 and the antitoxin function of rOSBGs may co-contributed to the protection.

In our previous study, the immunogenicity and protection effect of recombinant Stx2Am-Stx1B has been detected, could induce cross-neutralizing antibodies and effective protection^13^. Many similar constructs using the A or B subunit of Shiga toxin (Stx) elicited both bactericidal and toxin-neutralizing antibodies in mice. So far there is no clinical study of Stx-based human vaccine. In this study, we aimed to develop a new high effective oral vaccine against EHEC infection, which may easy accept to human. And Bacterial ghost (BG) based candidate vaccine have been used in various pathogen^23^,^24^,^25^,^26^,^27^, which show high sevurity and effectiveness. Thus, rOSBGs may be a better choice to children against EHEC.

Attaching/effacing and Shiga toxins are two important factors in infection of *E. coli* O157:H7. The co-infection of viable organisms and toxins is the general case in clinic examination^46^. In this study, the histopathology also revealed that the challenge of lysed O157:H7 caused damage of kidney (Stxs lesions), and the challenge of viable O157:H7 caused attaching and effacing lesions. *E. coli* O157:H7 infection characteristically results in attaching and effacing (A/E) lesions and toxin-induced systemic injury. Therefore, the novel vaccine candidate rSOBGs, which could provide co-prevention against the adhesion of bacteria and damage of Stxs, is a promising vaccine in human.

## Additional Information

**How to cite this article**: Cai, K. *et al*. Novel fusion antigen displayed-bacterial ghosts vaccine candidate against infection of Escherichia coli O157:H7. *Sci. Rep*. **5**, 17479; doi: 10.1038/srep17479 (2015).

## Supplementary Material

Supplementary Information

## References

[b1] Karmali M. A. Infection by Shiga Toxin-Producing Escherichia coli: an overview. Mol. Biotechnol. 26, 117–122 (2004). 14764937 10.1385/MB:26:2:117

[b2] Tarr P. I., Gordon C. A. & Chandler W. J. Shiga-toxin-producing Escherichia coli and haemolytic uraemic syndrome. Lancet 365, 1073–1086 (2005). 15781103 10.1016/S0140-6736(05)71144-2

[b3] Centers for Disease Control and Prevention. Addressing Emerging Infectious Disease Threats: A Prevention Strategy for the United States Executive Summary. Morb. Mortal. Wkly. Rep. 43, 1–18 (1994). 8164632

[b4] Paton J. C. & Paton A. W. Pathogenesis and diagnosis of Shiga toxin-producing Escherichia coli infections. Clin. Microbiol. Rev. 11, 450–479 (1998). 9665978 10.1128/cmr.11.3.450 PMC88891

[b5] Fraser M. E., Chernaia M. M., Kozlov Y. V. & James M. N. Crystal structure of the holotoxin from Shigella dysenteriae at 2.5 A resolution. Nat. Struct. Biol. 1, 159–164 (1994). 10.1038/nsb0194-59 7656009

[b6] Fraser M. E. . Structure of Shiga toxin type 2 (Stx2) from Escherichia coli O157:H7. J. Biol. Chem. 279, 27511–27517 (2004). 15075327 10.1074/jbc.M401939200

[b7] Kaper J. B. & O’Brien A. D. Escherichia coli O157:H7 and other shiga toxin-producing E. coli strains. Washington, DC; ASM Press. (1998).

[b8] Acheson D. W. . Expression and purification of shiga-like toxin□B subunits. Infect. Immun. 63, 301–308 (1995). 7806370 10.1128/iai.63.1.301-308.1995 PMC172992

[b9] Wen S. X., Teel L. D., Judge N. A. & O’Brien A. D. Genetic toxoids of Shiga toxin types 1 and 2 protect mice against homologous but not heterologous toxin challenge. Vaccine 24, 1142–1148 (2006). 16198455 10.1016/j.vaccine.2005.08.094

[b10] Strockbine N. A. . Two toxin-converting phages from Escherichia coli O157:H7 strain 933 encode antigenically distinct toxins with similar biological activities. Infect. Immun. 53, 135–140 (1986). 3522426 10.1128/iai.53.1.135-140.1986 PMC260087

[b11] Smith M. J., Teel L. D., Carvalho H. M., Melton-Celsa A. R. & O’Brien A. D. Development of a hybrid Shiga holotoxoid vaccine to elicit heterologous protection against Shiga toxin types 1 and 2. Vaccine 24, 4122–4129 (2006). 16551486 10.1016/j.vaccine.2006.02.035

[b12] Gao X. . Immunogenicity of a novel Stx2B-Stx1B fusion protein in a mice model of Enterohemorrhagic Escherichia coli O157:H7 infection. Vaccine 27, 2070–2076 (2009). 19428832 10.1016/j.vaccine.2009.01.115

[b13] Cai K. . Enhanced immunogenicity of a novel Stx2Am-Stx1B fusion protein in a mice model of enterohemorrhagic Escherichia coli O157:H7 infection. Vaccine 29, 946–952. (2011). 21134452 10.1016/j.vaccine.2010.11.035

[b14] Konadu E. Y. . Investigational vaccine for Escherichia coli O157: phase 1 study of O157 O-specific polysaccharide-Pseudomonas aeruginosa recombinant exoprotein A conjugates in adults. Infect. Dis. 177, 383–387 (1998). 10.1086/514203 9466525

[b15] Judge N. A., Mason H. S. & O’Brien A. D. Plant cell-based intimin vaccine given orally to mice primed with intimin reduces time of E. coli O157:H7 shedding in feces. Infect. Immun. 72, 168–175 (2004). 14688094 10.1128/IAI.72.1.168-175.2004 PMC343997

[b16] Gansheroff L. J., Wachtel M. R. & O’Brien A. D. Decreased adherence of enterohemorrhagic Escherichia coli to HEp-2 cells in the presence of antibodies that recognize the C-terminal region of intimin. Infect. Immun. 67, 6409–6417 (1999). 10569757 10.1128/iai.67.12.6409-6417.1999 PMC97049

[b17] Potter A. A. . Decreased shedding of Escherichia coli O157:H7 by cattle following vaccination with type III secreted proteins. Vaccine 22, 362–369 (2004). 14670317 10.1016/j.vaccine.2003.08.007

[b18] Li Y., Frey E., Mackenzie A. M. & Finlay B. B. Human response to Escherichia coli O157:H7 infection: antibodies to secreted virulence factors. Infect. Immun. 68, 5090–5095 (2000). 10948130 10.1128/iai.68.9.5090-5095.2000 PMC101746

[b19] Funatogawa K. . Use of immunoglobulin enriched bovine colostrum against oral challenge with enterohaemorrhagic Escherichia coli O157:H7 in mice. Microbiol. Immunol. 46, 761–766 (2002). 12516772 10.1111/j.1348-0421.2002.tb02761.x

[b20] Witte A., Wanner G., Sulzner M. & Lubitz W. Dynamics of PhiX174 protein E-mediated lysis of Escherichia coli. Arch. Microbiol. 157, 381–388 (1992). 1534215 10.1007/BF00248685

[b21] Witte A. & Lubitz W. Biochemical characterization of PhiX174 protein E-mediated lysis of Escherichia coli. Eur. J Biocem. 180, 393–398 (1989). 10.1111/j.1432-1033.1989.tb14661.x 2522390

[b22] Riedmann E. M., Kyd J. M., Cripps A. W. & Lubitz W. Bacterial ghosts as adjuvant particles. Expert. Rev. Vaccines 6, 241–253 (2007). 17408373 10.1586/14760584.6.2.241

[b23] Eko F. O. . Evaluation of the protective efficacy of Vibrio cholerae ghost (VCG) candidate vaccines in rabbits. Vaccine 21, 3663–3674 (2003). 12922096 10.1016/s0264-410x(03)00388-8

[b24] Marchart J. . Protective immunity against pasteurellosis in cattle, induced by Pasteurella haemolytica ghosts. Vaccine 21, 1415–1422 (2003). 12615438 10.1016/s0264-410x(02)00635-7

[b25] Panthel K. . Generation of Helicobacter pylori ghosts by PhiX protein E-mediated inactivation and their evaluation as vaccine candidates. Infect. Immun. 71, 109–116 (2003). 12496155 10.1128/IAI.71.1.109-116.2003 PMC143412

[b26] Mayr U. B. . Bacterial ghosts as an oral vaccine: a single dose of Escherichia coli O157:H7 bacterial ghosts protects mice against lethal challenge. Infect. Immun. 73, 4810–4817 (2005). 16040994 10.1128/IAI.73.8.4810-4817.2005 PMC1201255

[b27] Cai K. . Intragastric immunization of mice with enterohemorrhagic Escherichia coli O157:H7 bacterial ghosts reduces mortality and shedding and induces a Th2-type dominated mixed immune response. Can. J .Microbiol. 56, 389–398 (2010). 20555401 10.1139/w10-025

[b28] Pistor S. & Hobom G. Expression of viral hemagglutinin on the surface of E. coli. Klin. wochenschr. 66, 110–116 (1988). 3280869 10.1007/BF01774224

[b29] Ruppert A., Arnold N. & Hobom G. OmpA-FMDV VP1 fusion proteins: production, cell surface exposure and immune responses to the major antigenic domain of foot-and-mouth disease virus. Vaccine 12, 492–498 (1994). 8036821 10.1016/0264-410x(94)90305-0

[b30] Schorr J., Knapp B., Hundt E., Küpper H. A. & Amann E. Surface expression of malarial antigens in Salmonella typhimurium: induction of serum antibody response upon oral vaccination of mice. Vaccine 9, 675–681 (1991). 1950099 10.1016/0264-410x(91)90194-b

[b31] Jechlinger W. . Comparative immunogenicity of the hepatitis B virus core 149 antigen displayed on the inner and outer membrane of bacterial ghosts. Vaccine 23, 3609–3617 (2005). 15855021 10.1016/j.vaccine.2004.11.078

[b32] Wang Q. . Passive protection of purified yolk immunoglobulin administered against Shiga toxin 1 in mouse models. Can. J. Microbiol. 56, 1003–1010 (2010). 21164570 10.1139/W10-087

[b33] Gao X. . Prokaryotic expression and immunogenicity analysis of intimin polypeptides of enterohemorrhagic Escherichia coli. Bull. Acad. Mil. Med. Sci. 32, 316–318 (2008).

[b34] Klose M., Jähnig F., Hindennach I. & Henning U. Restoration of membrane incorporation of an Escherichia coli outer membrane protein (OmpA) defective in membrane insertion. J. Biol. Chem. 264, 21842–21847 (1989). 2689448

[b35] Dornmair K., Kiefer H. & Jähnig F. Refolding of an integral membrane protein. OmpA of Escherichia coli. J. Biol. Chem. 265, 18907–18911 (1990). 2229053

[b36] Freudl R., Schwarz H., Klose M., Movva N. R. & Henning U. The nature of information, required for export and sorting, present within the outer membrane protein OmpA of Escherichia coli K-12. EMBO J. 4, 3593–3598 (1985). 3912172 10.1002/j.1460-2075.1985.tb04122.x PMC554702

[b37] Pistor S. & Hobom G. Expression of viral hemagglutinin on the surface of E. coli. Klin wochenschr 66, 110–116 (1988). 3280869 10.1007/BF01774224

[b38] Braun G. & Cole S. T. The nucleotide sequence coding for major outer membrane protein OmpA of Shigella dysenteriae. Nucleic. Acids. Res. 10, 2367–2378 (1982). 6283478 10.1093/nar/10.7.2367 PMC320615

[b39] Ruppert A., Arnold N. & Hobom G. OmpA-FMDV VP1 fusion proteins: production, cell surface exposure and immune responses to the major antigenic domain of foot-and-mouth disease virus. Vaccine 12, 492–498 (1994). 8036821 10.1016/0264-410x(94)90305-0

[b40] Schorr J., Knapp B., Hundt E., Küpper H. A. & Amann E. Surface expression of malarial antigens in Salmonella typhimurium: induction of serum antibody response upon oral vaccination of mice. Vaccine 9, 675–681 (1991). 1950099 10.1016/0264-410x(91)90194-b

[b41] Jechlinger W. . Comparative immunogenicity of the hepatitis B virus core 149 antigen displayed on the inner and outer membrane of bacterial ghosts. Vaccine 23, 3609–3617 (2005). 15855021 10.1016/j.vaccine.2004.11.078

[b42] Conlan J. W., Cox A. D., KuoLee R., Webb A. & Perry M. B. Parenteral immunization with a glycoconjugate vaccine containing the O157 antigen of Escherichia coli O157:H7 elicits a systemic humoral immune response in mice, but fails to prevent colonization by the pathogen. Can. J. Microbiol. 45, 279–286 (1999). 10420579

[b43] Ohmura M. . Nontoxic Shiga toxin derivatives from Escherichia coli possess adjuvant activity for the augmentation of antigen-specific immune responses via dendritic cell activation. Infect. Immun. 73, 4088–4097 (2005). 15972497 10.1128/IAI.73.7.4088-4097.2005 PMC1168555

[b44] Boerlin P. . Associations between virulence factors of Shiga toxin-producing Escherichia coli and disease in humans. J. Clin. Microbiol. 37, 497–503 (1999). 9986802 10.1128/jcm.37.3.497-503.1999 PMC84443

[b45] Robinson C. M., Sinclair J. F., Smith M. J. & O’Brien A. D. Shiga toxin of enterohemorrhagic Escherichia coli type O157:H7 promotes intestinal colonization. Proc. Natl. Acad. Sci. 103, 9667–9672 (2006). 16766659 10.1073/pnas.0602359103 PMC1475797

[b46] Gao X. . Novel fusion protein protects against adherence and toxicity of enterohemorrhagic Escherichia coli O157:H7 in mice. Vaccine 29, 6656–6663 (2011). 21742003 10.1016/j.vaccine.2011.06.106

