# Supplementary material for: Novel fusion antigen displayed-bacterial ghosts vaccine candidate against infection of Escherichia coli O157:H7
Source: Sci Rep. 2015 Dec 2;5:17479. doi: 10.1038/srep17479 (PMC4667225; doi:10.1038/srep17479)
Supplement: Supplementary Information [file srep17479-s1.doc]

Novel fusion antigen displayed-bacterial ghosts vaccine candidate against infection of Escherichia coli O157:H7

Kun Cai, Wei Tu, Yuenan Liu, Tao Li*, Hui Wang*

State Key Laboratory of Pathogens and Biosecurity, Beijing Institute of Microbiology and Epidemiology, No. 20 Dongdajie, Fengtai District, Beijing 100071, PR China.

* Corresponding author. Beijing Institute of Microbiology and Epidemiology. No. 20 Dongdajie, Fengtai District, Beijing 100071, PR China.Tel.: +86 01066948532; E-mail address: [geno0109@vip.sina.com](mailto:geno0109@vip.sina.com)(H. Wang). Additional corresponding author. Tel.: +86 01066948587; E-mail address: [litaobmi@126.com](mailto:litaobmi@gmail.com) (T. Li).

**Supp. Legends**

Fig.1. Schematic diagrams of the rSOBG and recombinant plasmids.

1. The potential spatial structure of the rSOBG. ○: Stx2Am；△: Stx1B；-：GGGGS.
2. B. The construction of the lysed plasmid pLysE and displayed plasmid pOSAmB.

Fig 1A


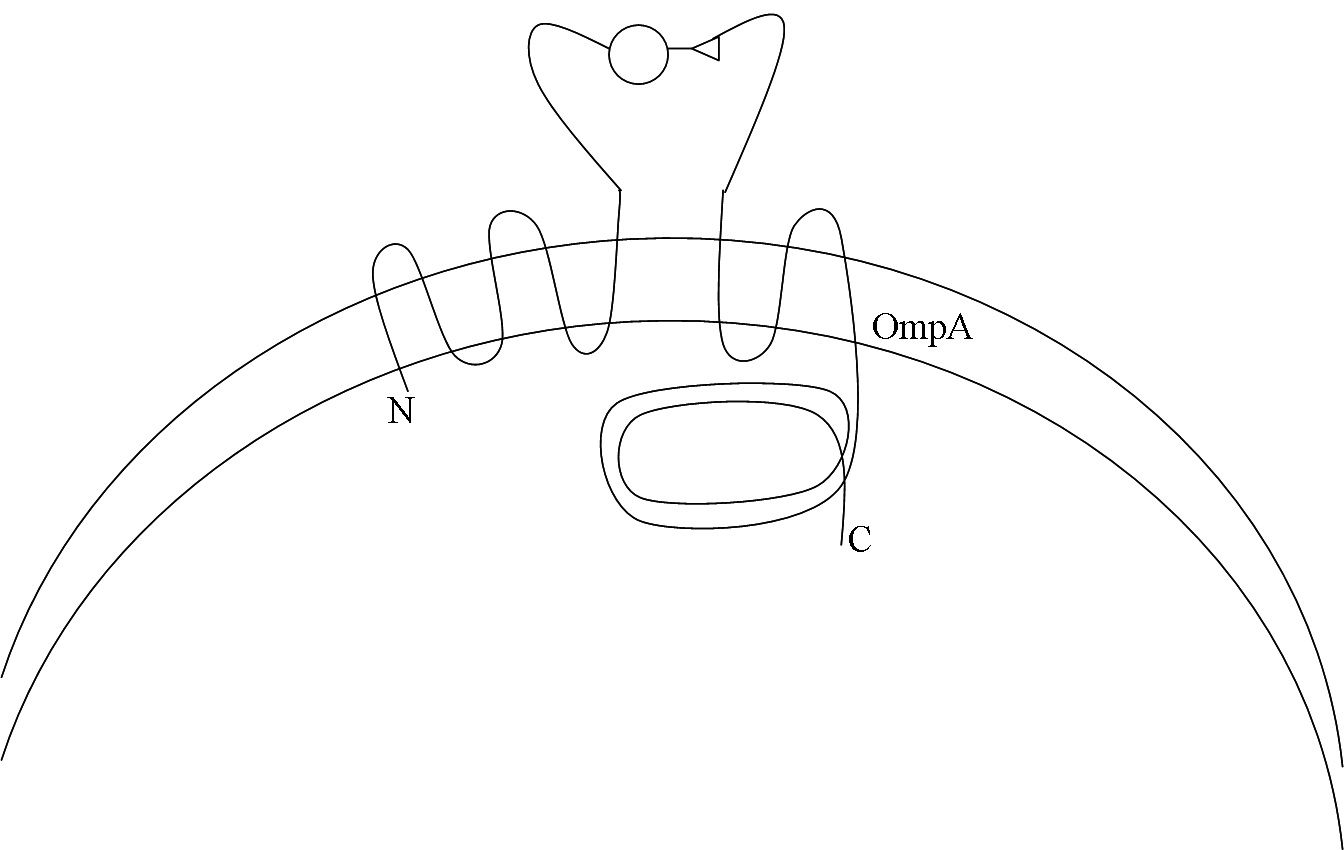


Fig 1B

Table S1 Synthetic oligonucleotide primers used in this study

| Primer | Sequence (5’-3’) ^*^ | Region of homology  or Purpose |
| --- | --- | --- |
| Stxup | GAATTCCGGGAGTTTACGATAGACTTTTCGACCCAA | Stx upstream primer |
| Stxdown | GAGCTCACGAAAAATAACTTCGCTGAATCCCCC | Stx downstream primer |
| SOup | CCCGGGATGAAAAAGACAGCTATCGCGATTACA | Shigella dysenteriae OmpA upstream primer |
| SOdown | ACCCAGAACAACTACGGAACCGTCTTTCGG | E.coli OmpA downstream primer |
| EOup | CCGAAAGACGGTTCCGTAGTTGTTCTGGGT | Shigella dysenteriae OmpA upstream primer |
| EOdown | CCATGGTTAAGCTTGCGGCTGAGTTACAAC | E.coli OmpA downstream primer |
| Enzup | CACAACAATGTGACAGGTGAAGAATTCATCGATACTTGATCTAAGATATCATCATTA | Restriction enzyme site upstream primer |
| Enzdown | TCGTGGTTTTTCTCAGAGAGCTCACTAGTTAATGATGATATCTTAGATCAAGT | Restriction enzyme site downstream primer |

*. The underline place is restriction enzymes

Table S2 the groups of mice immunization and challenge in the study.

| Groups | | Mice Number | Antigen | Objective |
| --- | --- | --- | --- | --- |
| A | A1 | 25 | rSOBGs | Intragastric challenge of 100 LD50 (109 CFU) viable E. coli O157:H7 EDL933 |
|  | A2 | 25 |  | Intragastric challenge of 500 LD50 viable E. coli O157:H7 EDL933 |
|  | A3 | 15 |  | Intraperitoneal injection with 2 LD50 (5×10^8^ CFU) lysed E. coli O157:H7 88321 |
|  | A4 | 15 |  | Intraperitoneal injection with 5 LD50 lysed E. coli O157:H7 88321 |
|  | A5 | 15 |  | Collect serum and irrigating solution of intestinal tract on days 0, 7, 14, 21 and 28 |
|  | A6 | 10 |  | Collect the sera 14 days post the last immunization |
| B | B1 | 25 | OBGs | Intragastric challenge of 100 LD50 (109 CFU) viable E. coli O157:H7 EDL933 |
|  | B2 | 25 |  | Intragastric challenge of 500 LD50 viable E. coli O157:H7 EDL933 |
|  | B3 | 15 |  | Intraperitoneal injection with 2 LD50 (5×10^8^ CFU) lysed E. coli O157:H7 88321 |
|  | B4 | 15 |  | Intraperitoneal injection with 5 LD50 lysed E. coli O157:H7 88321 |
|  | B5 | 15 |  | Collect serum and irrigating solution of intestinal tract on days 0, 7, 14, 21 and 28 |
|  | B6 | 10 |  | Collect the sera 14 days post the last immunization |
| C | C1 | 25 | PBS | Intragastric challenge of 100 LD50 (109 CFU) viable E. coli O157:H7 EDL933 |
|  | C2 | 25 |  | Intragastric challenge of 500 LD50 viable E. coli O157:H7 EDL933 |
|  | C3 | 15 |  | Intraperitoneal injection with 2 LD50 (5×10^8^ CFU) lysed E. coli O157:H7 88321 |
|  | C4 | 15 |  | Intraperitoneal injection with 5 LD50 lysed E. coli O157:H7 88321 |
|  | C5 | 15 |  | Collect serum and irrigating solution of intestinal tract on days 0, 7, 14, 21 and 28 |
|  | C6 | 10 |  | Collect the sera 14 days post the last immunization |
